# Rescue medical activities in the mediterranean migrant crisis

**DOI:** 10.1186/s13031-017-0105-1

**Published:** 2017-03-22

**Authors:** Favila Escobio, Maryse Etiennoul, Stephany Spindola

**Affiliations:** Medicines du Monde France, Mediterranean Search and Rescue Project, Rue Marcadet 62, Paris, 75018 France

The central Mediterranean route, between Libya and Italy, is considered the most dangerous of the migration pathways to Europe. In 2015, 3771 people died trying to reach Europe’s shores; and there were 4655 deaths or disappearances between January and November 2016 [1]. In response to this extreme situation, in early 2016, Medicines du Monde France (MdM), in partnership with SOS Mediterranee, launched an emergency project on board of the MV Aquarius, a ship adapted for search and rescue operations. We describe here the main clinical features observed during search and rescue activities in the central Mediterranean route. Existing studies present medical activities for migrants upon disembarkation, whereas there is far less information on the medical conditions during rescue operations [2, 3].

We set up a clinic on the Aquarius to provide emergency medical care. In addition, psychological first aid, emergency shelter and information services were also provided by the MdM team. The team included two physicians, two nurses, one logistician, one communication officer and one interpreter. SOS Mediterranee search and rescue members were also trained by the MdM medical team to identify severe conditions and provide first aid during rescue. A medical doctor was always available on standby during the approach and rescue manoeuvres. A visual assessment and triage based on the South African Triage Score (SATS) were conducted on the deck by a medical staff member immediately after people were secured on board [4]. Severely sick and injured patients remained in the clinic for observation and follow-up. Decisions regarding medical evacuation and referral upon disembarkation were based on case severity and vulnerability, using the physicians’ clinical assessments and resources available on board.

Demographic and clinical data were collected during the intervention by MdM medical staff. During the operation between February and May 2016, out of the 919 people rescued, 212 medical consultations were provided by the medical team on board. All people were rescued from inflatable boats which departed from Libya and the main countries of origin reported by patients were Gambia (27.8%), Nigeria (24.1%) and Senegal (11.8%). Unaccompanied male minors were the main group in our medical consultations (43.6%), followed by both male (35.1%) and female (21.3%) adults.

The most frequent medical conditions were accidental trauma (24.1%), medically unexplained physical symptoms (14.2%), intentional trauma (6.6%) and gastrointestinal problems (6.6%). These were often also accompanied by symptoms of mild and moderate hypothermia. The main causes of accidental trauma were chemical burns due to benzene (52.9%), contusions (25.6%) and wounds (21.5%).The main causes of intentional trauma were contusions (50%), bullet injuries (28.5%) and wounds (21.4%).Nearly a third (31%) of patients who attended the clinic had reported recent exposure to violent events. It was a challenge to properly screen mental health conditions and to fully identify severe psychological trauma cases due to time and space constraints on board.

The lack of security and physical protection during the travel in overcrowded and unsafe inflatable boats magnifies the presence of accidental trauma, while gastrointestinal problems and symptoms of mild and moderate hypothermia can be associated with the changing weather conditions at sea. Medically unexplained physical symptoms are likely to be related to traumatic events during the travel, including witnessing people drowning, along with previous repeated exposure to violent events such as sexual violence, kidnapping and human trafficking, which all contribute to the burden of intentional trauma-related conditions and mental disorders among the rescued people [5].

This letter provides a snapshot of the clinical features of rescued people.It highlights the substantial health risks among migrants using the central Mediterranean route and the need for adequate health service responses, including during search and rescue operations. MdM continues addressing these urgent health needs and helps to ensure safe routes and access to universal health care for those fleeing conflict, war and poverty [6, 7].

## References

[1] IOM Mediterranean update report. Migration Flows Europe: Arrivals and Fatalities. https://www.iom.int/news/mediterranean-migrant-arrivals-reach-345440-deaths-sea-4655 (Accessed date 7 Dec 2016).

[2] Trovato A, Reid A, Takarinda KC (2016). Dangerous crossing: demographic and clinical features of rescued sea migrants seen in 2014 at an outpatient clinic at Augusta Harbor, Italy. Confl Heal.

[3] Kulla M, Josse F, Stierholz M, Hossfeld B, Lampl L, Helm M (2016). Initial assessment and treatment of refugees in the Mediterranean Sea (a secondary data analysis concerning the initial assessment and treatment of 2656 refugees rescued from distress at sea in support of the EUNAVFOR MED relief mission of the EU). Scand J Trauma Resusc Emerg Med.

[4] Sunyoto T, Van den Bergh R, Valles P (2014). Providing emergency care and assessing a patient triage system in a referral hospital in Somaliland: a cross-sectional study. BMC Health Serv Res.

[5] The illness of migration, ten years of medical humanitarian assistance to migrants in Europe and in transit countries. https://www.aerzte-ohne-grenzen.de/sites/germany/files/attachments/msf-the-illness-of-migration-2013.pdf (Accessed 3 Nov 2016).

[6] Frank Vanbiervliet, Nathalie Simonnot. MdM position on migrants in transit. Final version 20th January 2016. https://mdmeuroblog.files.wordpress.com/2016/07/en-mdm-network-position-paper-on-migrant-reception-crisis.pdf (Accesed 3 Nov 2016).

[7] Humanitarian Visa. Appel de Neuchatel. June 2016. http://www.humanitarianvisa.org/wp-content/uploads/2016/06/DOSSIER-DE-PRESSE-21-JUIN-16_embargo-21-juin-%C3%A0-16-h-00-1.pdf (Accessed 3 Nov 2016).

